# Host, technical, and environmental factors affecting QuantiFERON-TB Gold In-Tube performance in children below 5 years of age

**DOI:** 10.1038/s41598-022-24433-w

**Published:** 2022-11-19

**Authors:** Eneritz Velasco-Arnaiz, Marta Batllori, Manuel Monsonís, Anna Valls, María Ríos-Barnes, Sílvia Simó-Nebot, Anna Gamell, Clàudia Fortuny, Marc Tebruegge, Antoni Noguera-Julian

**Affiliations:** 1 Malalties Infeccioses i Resposta Inflamatòria Sistèmica en Pediatria; Servei d’Infectologia Pediàtrica, Institut de Recerca Sant Joan de Déu, Barcelona, Spain; 2 Laboratori de Bioquímica, Hospital Sant Joan de Déu, Barcelona, Spain; 3 Servei de Microbiologia. Hospital Sant Joan de Déu, Barcelona, Spain; 4 CIBER de Epidemiología y Salud Pública (CIBERESP), Madrid, Spain; 5 Departament de Cirurgia i Especialitats Medicoquirúrgiques, Facultat de Medicina i Ciències de la Salut, Universitat de Barcelona, Barcelona, Spain; 6 Translational Research Network in Pediatric Infectious Diseases (RITIP), Madrid, Spain; 7 Department of Infection, Immunity and Inflammation, UCL Great Ormond Street Institute of Child Health, University College London, London, UK; 8 Department of Pediatrics, University of Melbourne, Parkville, Australia; 9 Department of Paediatrics, Klinik Ottakring, Wiener Gesundheitsverbund, Vienna, Austria; 10 Hospital Sant Joan de Déu, Passeig Sant Joan de Déu 2, Esplugues de Llobregat, 08950 Barcelona, Spain

**Keywords:** Immunology, Infectious diseases

## Abstract

Interferon-gamma release assays performance can be impaired by host-related, technical and environmental factors, but data in young children are limited. We performed a cross-sectional study of children < 5 years-of-age at risk of tuberculosis (TB), using QuantiFERON-TB Gold In-Tube (QFT-GIT) assays. The impact of the following was evaluated: (i) host-related [age; hematological parameters; erythrocyte sedimentation rate (ESR); C-reactive protein (CRP); and tobacco smoke exposure (TSE) based on serum cotinine concentrations], (ii) technical (pre-analytical delay) and (iii) environmental factors (annual season; monthly temperatures). Of 204 children, 35 (17.2%) were diagnosed with latent TB infection or TB disease. QFT-GIT results were indeterminate in 14 (6.9%) patients. In multivariate analysis, younger age and higher ESR were associated with lower positive control responses (beta: 0.247, *p* = 0.002 and − 0.204, *p* = 0.007, respectively), and increasing age was associated with lower rates of indeterminate QFT-GIT results [OR (95% CI) 0.948 (0.903–0.996) per month, *p* = 0.035]. In children with positive QFT-GIT results, average monthly temperatures correlated with antigen responses (r = 0.453, *p* = 0.020); also, antigen responses were lower in winter than in other seasons (*p* = 0.027). Serum cotinine concentrations determined in a subgroup of patients (n = 41) indicated TSE in 36 (88%), positive control responses being lower in children with TSE (*p* = 0.034). In children < 5 years-of-age, young age, elevated ESR, temperature, annual season and TSE can affect the performance of QFT-GIT assays.

## Introduction

Interferon-gamma (IFN-γ) release assays (IGRAs) are an alternative to the tuberculin skin test (TST) for the diagnosis of latent tuberculosis (TB) infection (LTBI) and TB disease, but increasing evidence suggests that their performance is not superior to the latter, particularly in infants and young children^1^. The currently most widely used IGRA in routine clinical practice is the QuantiFERON-TB Gold assay (Cellestis/Qiagen, Carnegie, Australia)^2,3^.

Most guidelines include IGRAs as complementary tools to increase sensitivity, but provide little guidance on how to interpret discordance between TST and IGRA results, as well as IGRA result conversion and reversion^4–8^. A further significant issue in clinical practice are indeterminate IGRA results, which have been reported to occur in 0.6–35% of pediatric patients^9,10^. Previous data, including our own, show that the rate of indeterminate results is particularly high in children below 2 years of age^11^, most commonly resulting from insufficient mitogen (positive control) responses, which has been attributed to immunological immaturity^12–15^. Young age also appears to increase the risk of false-negative IGRA results^16^. Furthermore, previous data suggest the diagnostic accuracy of IGRAs can be impaired by a range of other host-related, technical and environmental factors^17–19^.

In adults, cigarette smoking has been associated with a reduction in IFN-γ production and higher rates of false-negative and indeterminate QFT-GIT results^20,21^. However, to date, the impact of tobacco smoke exposure (TSE) on IGRA results in children has not been investigated. Cotinine, a major metabolite of nicotine, has been used to assess tobacco use and environmental exposure both in active and passive smokers^22,23^. The half-life of cotinine in plasma is longer than that of nicotine. The former is estimated to be around 16 h in adults, but longer in neonates, infants, and children, thereby reflecting exposure over the preceding days^24–26^.

This study aimed to investigate the impact of a range of host-related factors on QFT-GIT assay performance specifically in children below 5 years of age in routine clinical practice in a low TB prevalence setting. Secondary objectives included the evaluation of other technical and environmental variables, including TSE for the first time, as well as the prevalence and risk factors of indeterminate QFT-GIT results.

## Results

In total, 204 children (104 females, 51.0%; median [IQR] age at assessment: 26 [11–40] months) were included (Table 1). Most were born in Spain (83.3%), were from immigrant families (59.8%), and were assessed for TB as part of contact tracing (65.7%). Regarding their final diagnosis, 169 (82.8%) were considered uninfected, while 11 (5.4%) and 24 (11.8%) were diagnosed with LTBI and TB disease (microbiologically-confirmed in 9/24, 37.5%). Indeterminate QFT-GIT results were observed in 14/204 (6.9%) children; all were due to insufficient positive control (mitogen) responses (Supplementary Tables S1 and S2).

**Table 1:** Impact of host-related, technical, and environmental factors on the rate of determinate (i.e. positive or negative) and indeterminate QFT-GIT assay results. Data shown as number (percentage) or median (interquartile range).

	QFT-GIT		*p-*value
	Determinate result n = 190 (93.1)	Indeterminate result n = 14 (6.9)	
**Host factors**			
Gender, female	98 (51.6)	6 (42.9)	0.362
Age (months)	27 (12–40)	14 (6–22)	**0.014**
Immigrant child	32 (16.8)	2 (14.3)	0.578
Immigrant family	115 (60.5)	7 (50.0)	0.307
BCG-vaccinated	16 (8.4)	0	0.339
Hb (g/dL)^a^	12.2 (11.4–12.7)	12.1 (10.9–12.4)	0.515
Low Hb			
DAIDS Grade 1	10/174 (5.7)	2/11 (18.2)	0.437
DAIDS Grade 2	6/174 (3.4)	0	
DAIDS Grade 3	3/174 (1.7)	0	
DAIDS Grade 4	0	0	
Platelets (cells/mL)^a^	376.0 (312.5–440.5)	370.0 (322.0–532.0)	0.800
Low platelet counts			
DAIDS Grade 1	0	0	0.972
DAIDS Grade 2	1/174 (0.6)	0	
DAIDS Grade 3/4	0	0	
High platelet counts	37/174 (21.2)	3/11 (27.3)	0.446
WBC (cells/mL)^a^	9.7 (7.4–12.0)	11.2 (10.0–14.0)	0.090
Low WBC			
DAIDS Grade 1–4	0	0	–
ALC (cells/mL)	4.3 (3.2–6.1)	5.4 (3.4–6.9)	0.517
Abnormal ALC			0.543
Low ALC	12/174 (6.9)	1/11 (9.1)	
High ALC	17/174 (9.8)	0	
ANC (cells/mL)	3.3 (2.5–4.7)	4.0 (2.7–5.4)	0.384
Low ANC			
DAIDS Grade 1	3/174 (1.7)	0	0.878
DAIDS Grade 2–4	1/174 (0.6)	0	
AMC (cells/mL)	0.5 (0.4–0.7)	0.9 (0.5–1.4)	**0.023**
Abnormal AMC			**0.033**
Low AMC	0	0	
High AMC	42/174 (24.1)	6/11 (54.5)	
NLR	0.76 (0.51–1.16)	0.63 (0.43–1.48)	0.972
MLR	0.12 (0.09–0.17)	0.19 (0.10–0.22)	**0.047**
PLR	84.0 (63.5–114.0)	74.0 (55.0–96.0)	0.491
Eosinophils (cells/mL)	0.3 (0.2–0.4)	0.3 (0.1–0.4)	0.343
High eosinophil counts	25/174 (14.4)	0	0.365
ESR (mm)^a^	4 (2–10)	8 (2–18)	0.169
Elevated ESR	21/151 (13.9)	3/11 (27.3)	0.210
CRP (mg/L)^a^	1.0 (0.2–4.3)	11.3 (1.3–20.3)	**0.004**
Elevated CRP	18/153 (11.8)	3/11 (27.3)	0.152
**Technical factors**			
Pre-analytical delay (days)	7.0 (3.0–10.0)	5.5 (1.7–10.2)	0.544
**Environmental factors**			
Season of the year			0.715
Winter	56 (94.9)	3 (5.1)	
Spring	65 (94.2)	4 (5.8)	
Summer	36 (92.3)	3 (7.7)	
Autumn	33 (89.2)	4 (10.8)	
Temperature (°C)^b^	14.7 (11.2–19.7)	17.0 (11.7–19.1)	0.847
0–10	38 (92.7)	3 (7.3)	0.113
11–20	106 (93.0)	8 (7.0)	
21–30	46 (93.9)	3 (6.1)	
Cotinine (ng/mL)^c^	0.83 (0.20–1.90)	0.77 (0.66–0.77)	0.869
TSE			0.593
None/minimal	5/38 (13.2)	0	
Low	28/38 (73.6)	3/3 (100.0)	
High	5/38 (13.2)	0	

*Hb* hemoglobin level, *WBC* white blood cell count, *ALC* absolute lymphocyte count, *AMC* monocyte count, *ANC* neutrophil count, *NLR* neutrophil/lymphocyte ratio, *MLR* monocyte/lymphocyte ratio, *PLR* platelet/lymphocyte ratio, *ESR* erythrocyte sedimentation rate, *CRP* C-reactive protein, *TSE* tobacco smoke exposure.

^a^Patient numbers for Hb, platelet and white cell counts: patients with determinate QFT-GIT results, n = 172; with indeterminate results, n = 12. Numbers for ESR and CRP: patients with determinate QFT-GIT results, n = 152; with indeterminate results, n = 12.

^b^Median (IQR) temperatures per season (°C): winter, 10.0 (9.0–11.1); spring, 16.2 (13.5–19.3); summer 22.6 (21.7–24.9); and autumn, 16.7 (12.6–19.5).

^c^Patient numbers for cotinine concentration and TSE: patients with determinate QFT-GIT results, n = 38; with indeterminate results, n = 3.

In children with positive QFT-GIT results, background-corrected IFN-γ concentrations after TB antigen-stimulation showed no significant correlation with age, hematological variables including WBC ratios, CRP concentration, ESR values, cotinine levels or pre-analytical delay. However, there was a positive correlation between average monthly temperatures (r = 0.453, *p* = 0.020) and background-corrected IFN-γ concentrations (Supplementary Fig. S3). Also, in patients with positive QFT-GIT results, median background-corrected IFN-γ concentrations differed significantly between the seasons during which the tests were performed (winter: 1.41 [1.15–8.56], spring: 6.76 [3.90–9.98], summer: 10.99 [10.19–11.62] and autumn: 11.20 [8.56–13.04] IU/mL; p = 0.027) (Supplementary Fig. S4).

### Effect of factors studied on QFT-GIT positive control responses and indeterminate result rates

**Figure 1:**
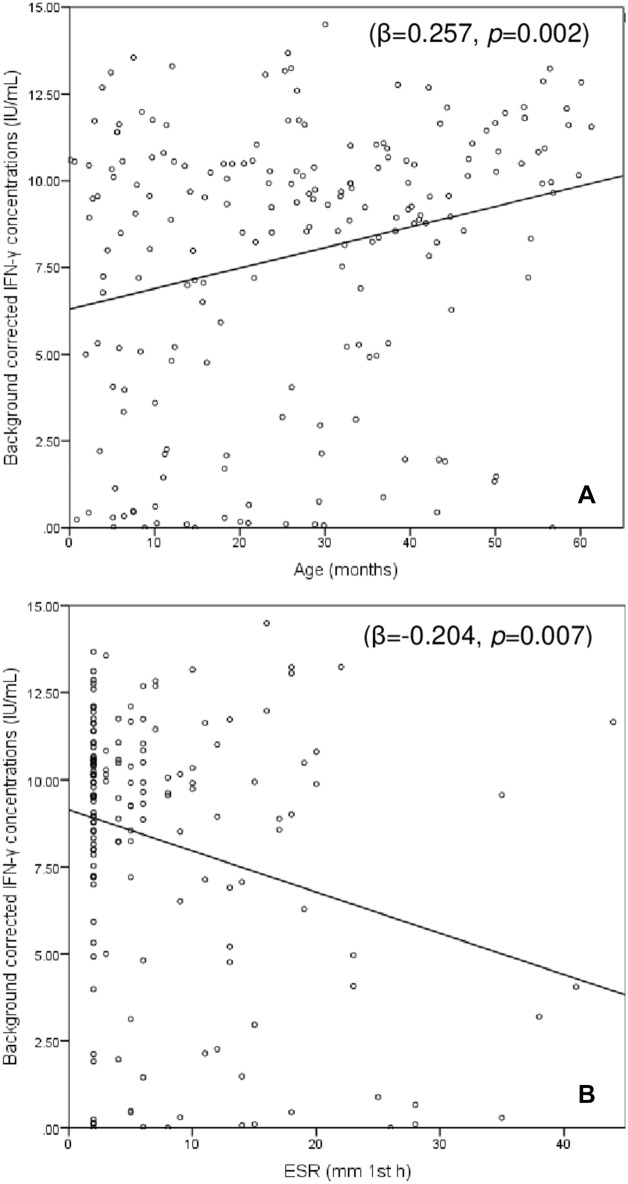
Correlation between background-corrected IFN-γ concentrations in the QFT-GIT positive control tube and (**A**) age and (**B**) ESR values. The values shown are the beta standardized coefficients and the corresponding p value.

In the entire cohort, background-corrected IFN-γ concentrations following mitogen stimulation showed weak positive correlations with age (r = 0.231, *p* = 0.001), Hb level (r = 0.205, *p* = 0.005), and eosinophil count (r = 0.157, *p* = 0.035), weak inverse correlations with ESR (r = − 0.163, *p* = 0.040) and CRP concentrations (r = − 0.257, *p* = 0.001) and no associations with the rest of variables. In multivariate linear regression analysis that included age, eosinophil counts, CRP and ESR values, only age and ESR values remained significantly associated with background-corrected mitogen-induced IFN-γ concentrations (Fig. 1).

In bivariate analysis, age, AMC, MLR, and CRP values differed significantly between children with determinate and those with indeterminate QFT-GIT results (Table 1). Pre-analytical delay and the median temperature of the month did not have an impact on mitogen responses or the rate of indeterminate results (data not shown), and the rate of indeterminate results did not differ significantly between the seasons of the year (winter 5.1%, spring 5.8%, summer 7.7% and autumn 10.8%; *p* = 0.715). In binary logistic regression analysis including age, MLR, and CRP, only increasing age remained independently associated with a lower rate of indeterminate QFT-GIT results (OR [95% CI] 0.948 [0.903–0.996] per month of age, *p* = 0.035).

### TSE levels and their impact on QFT-GIT performance

Serum cotinine concentrations were measured in 41 children. The distribution of demographic and clinical characteristics of this subgroup was not significantly different from the whole study cohort (data not shown). QFT-GIT results were determinate in 38 (92.7%; positive n = 16, negative n = 22) and indeterminate in 3 (7.3%) of those children. Most children (36/41, 87.8%) had cotinine levels indicating TSE. However, there were no significant differences in cotinine levels between the three diagnostic groups (Supplementary Table S5).

**Figure 2:**
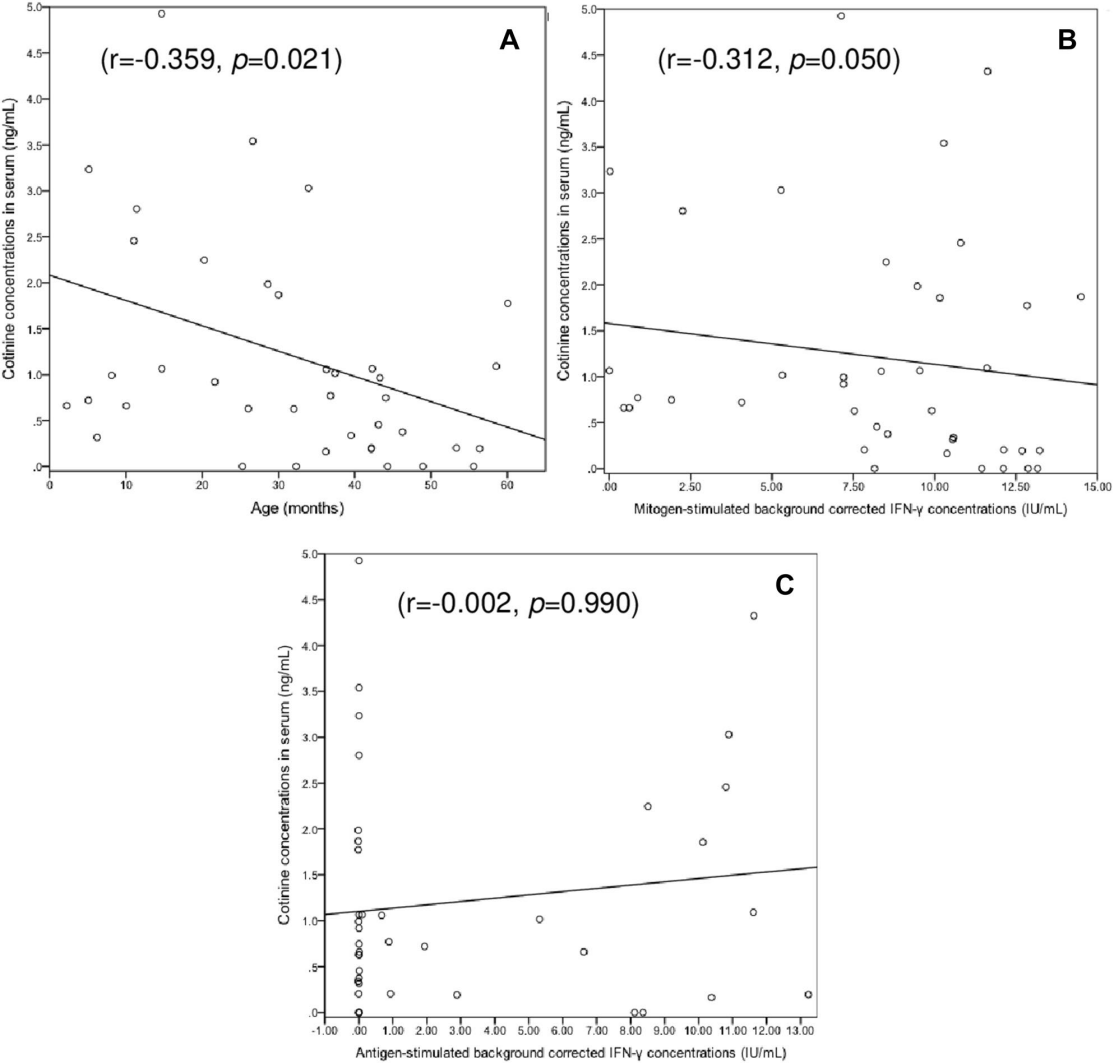
Correlation between serum cotinine concentrations and (**A**) age, (**B**) background-corrected IFN-γ concentrations in the QFT-GIT positive control tube, and (**C**) background-corrected IFN-γ concentrations in the QFT-GIT antigen-stimulated tube. The values shown are the Spearman correlation coefficient (r) and the corresponding *p* value.

Serum cotinine concentrations correlated inversely with age (r = − 0.359, p = 0.021) and background-corrected mitogen-induced IFN-γ concentrations (r = − 0.312, p = 0.050) (Fig. 2). On average the latter were significantly higher in children with no/minimal TSE than in those with low or high TSE (12.11 [9.80–13.01] vs. 8.51 [5.28–10.58] IU/mL; p = 0.034). However, the extent of TSE did not differ significantly between children with determinate QFT-GIT results and those with indeterminate results (Table 1).

## Discussion

To our knowledge, this is one of the largest studies of young children at risk of TB to analyze the impact of a broad range of several host-related, technical, and environmental factors on the performance of IGRAs in a high-income, low TB prevalence setting, and the first exploratory study investigating the impact of TSE on IGRAs in children below 5 years of age at risk of TB.

The proportion of indeterminate results in our cohort (6.9%) was consistent with the proportions reported by other pediatric studies^3,17,27^. In line with other studies reporting that the large majority of indeterminate results in children are due to insufficient positive control responses rather than failed nil controls, we found that all indeterminate results in our study were due to the former. Lower age was significantly associated with indeterminate QFT-GIT results in logistic regression analysis, and multivariate linear regression analysis showed a significant correlation between lower age and lower background-corrected mitogen-induced IFN-γ concentrations. These findings are in concordance with previous studies that have shown that indeterminate IGRA results are significantly more common in young children than in adolescents or middle-aged adults^3,17,27,28^. Similar to our observations, other pediatric studies also found that there is a positive correlation between age and the magnitude of positive control responses, likely reflecting evolving immune maturation over the first few years of life^27,28^. However, in contrast to positive control responses, TB antigen-induced IFN-γ responses appear to be age-independent^3^. These data suggest that in order to reduce the rate of indeterminate assay results in infants and toddlers, either age-specific cut-offs for positive control responses or alternative positive control stimulants should be used^3,27^.

Comparisons between children with determinate and those with indeterminate results using bivariate analysis showed that the latter had higher AMCs, MLRs, and CRP values, indicating that there may be an association between systemic inflammation and indeterminate test results. However, inflammatory parameters were within the normal range or only mildly elevated in most patients in our cohort and only the association between ESR values and the magnitude of mitogen responses in QFT-GIT remained significant in multivariate analysis. This aligns with recent studies showing that inflammation impairs IGRAs performance, both in healhy children and in those affected with immune-mediated inflammatory disorders^29–31^.

Previous data suggest that indeterminate IGRA results are more common during colder months (i.e. during autumn and winter) than in warmer months (i.e. during spring and summer)^19^, which we did not observe, potentially due to the limited size of our cohort. In contrast to the aforementioned study^19^, all IGRA samples were taken and processed within the same building, thereby limiting the potential impact of environmental temperatures, which have been shown to impact on QFT-GIT assay performance^18^. However, we found that TB antigen-induced IFN-γ responses were lower during winter than any of the other seasons. Given our study set-up it is very unlikely that environmental temperatures are the cause of this observation. A more likely explanation lies in seasonal variations in the functioning of the human immune system, which have been reported previously^32^.

The observed TSE was surprisingly high, especially considering that children are particularly susceptible to adverse consequences related to SHS exposure, including an increased risk of lower respiratory infections and severe asthma^23^. The information regarding SHS exposure in infants is currently scarce. In a study conducted in San Francisco in which 70% of the participants were < 3 years of age, more than 55% showed evidence of smoke exposure, based on plasma cotinine measurements^33^. Smoking is also an independent risk factor for TB infection, progression of LTBI to TB disease, and death from TB^34,35^. There is some evidence to suggest that smoke exposure impacts adversely on inflammatory and immune responses, including IFN-*γ* mediated signaling, which may impair QFT-GIT performance in adult smokers^21,36^. In our cohort higher cotinine concentrations were associated with lower IFN-*γ* responses after mitogen stimulation, although the rate of indeterminate results did not vary between the TSE categories used in this study. However, it is possible that the number of patients in whom cotinine levels were determined in our study was too small to detect relatively subtle, but potentially relevant differences.

Our study has a number of limitations, including the limited number of children diagnosed with LTBI or TB, and the lack of inclusion of other clinical (i.e. nutritional status or atopic background) and biochemical (i.e. ferritin, albumin or vitamin D levels) parameters that have been reported to potentially interfere with QFT-GIT performance. Furthermore, we did not collect any parental reports regarding smoking and their child’s SHS exposure; however, patient/parental reporting is deemed to be less accurate than biochemical testing^33^. Also, the design of the study prevents us from determining the impact of delays between blood draw and incubation of the samples^37,38^, as all blood samples were incubated in less than 90 min after phlebotomy.

In conclusion, in this cohort of previously healthy children below 5 years of age at risk of TB in a low prevalence setting, the rate of indeterminate QFT-GIT results was far higher than that reported by most adult studies, but consistent with other pediatric studies. Our observations show that indeterminate results are associated with lower age, and that both lower age and higher ESR values are associated with lower positive control responses. In contrast, none of the other factors investigated in this study, including blood cell counts and their derived ratios (NLR, MLR, and PLR), pre-analytical delay, and environmental factors, were found to have a significant impact on the performance of QFT-GIT assays. The impact of smoke exposure on IGRAs performance remains to be determined.

## Methods

### Study population

We performed a cross-sectional study in a cohort of previously healthy children below 5 years of age at risk of TB undergoing assessment between February 2012 and March 2016 at Hospital Sant Joan de Déu, a quaternary pediatric referral center in Barcelona, Spain. In Spain, the incidence of TB has persistently decreased over the past 20 years to 9.3 cases/100,000 in 2019 (4.2/100,000 in individuals aged < 15 years). Bacillus Calmette-Guérin (BCG) vaccination is not part of the routine childhood immunization program in Spain, except for the Basque Country, where a single dose was given at birth until 2013. Children were eligible if they were assessed due to clinical or radiological suspicion of TB, contact with a TB index case, or as part of new-entrant screening. The exclusion criteria comprised the following: previous diagnosis of LTBI or TB disease, pre-existing chronic medical conditions (including HIV infection) and/or current immunosuppressive therapy (including corticosteroids). This study was performed in accordance with the Declaration of Helsinki. Hospital Sant Joan de Déu ethics board approval was granted (ref. PIC 131-16); written informed consent was obtained from the parents/guardians of each participant prior to inclusion.

### Case definitions and follow-up

The diagnosis of TB disease was made based on epidemiological, clinical, radiological, and microbiological findings, independent of TST and QFT-GIT results, as previously described^1^. LTBI was defined as presence of a positive TST and/or QFT-GIT result in the absence of clinical and radiological signs of TB disease.

### TST and IGRA testing

TSTs were performed by intradermal injection of 0.1 ml (2TU) of purified protein derivative (Tuberkulin PPD RT23 SSI, Statens Serum Institut; Copenhagen, Denmark), with results being read after 48–72 h. Following current national Spanish guidelines, the cut-offs for a positive TST result were ≥ 5 mm of induration in children assessed for clinically and/or radiologically suspected TB disease and those assessed following TB contact, and ≥ 10 mm in children undergoing new-entrant screening, irrespective of BCG vaccination status^6,39^.

QFT-GIT (Cellestis/Qiagen; Carnegie, Australia) assays were performed and interpreted according to the manufacturer's instructions^40^. Blood samples were collected by a trained pediatric phlebotomist or by a pediatric nurse. Following shaking to ensure complete mixing of the blood sample with the tube additives, tubes were transported to an on-site, fully-accredited routine diagnostic laboratory in less than 90 min and were incubated immediately at 37 °C for 16 to 24 h. Then, tubes were centrifuged and plasma was removed and stored at 2–8 °C until the analysis was performed (every 1–2 weeks) measuring the amount of IFN-γ (in IU/mL) by enzyme-linked immunosorbent assay, after having brought the samples to room temperature (22 °C ± 5 °C). Briefly, a test was considered positive if the background-corrected IFN-γ response (i.e. concentration in TB-antigen tube minus concentration in the nil control tube) was ≥ 0.35 IU/mL and > 25% of the nil value. An indeterminate result was defined as insufficient IFN-γ response in the positive control tube (concentration < 0.5 IU/mL) and/or an IFN-γ concentration > 8.0 IU/mL in the nil control tube.

### Factors potentially affecting QFT-GIT assay performance

The following factors potentially affecting QFT-GIT performance and results were assessed:

i. Host-related: full blood-counts including hemoglobin (Hb) levels (g/dL), platelet counts (cells/mL), absolute and differential white blood cell counts (WBC), including absolute lymphocyte counts (ALC), monocyte (AMC) and neutrophil counts (ANC) (cells/mL; used to calculate monocyte-lymphocyte ratio [MLR], neutrophil-lympocyte ratio [NLR] and platelet-lymphocyte ratio [PLR] as surrogate markers of systemic inflammation)^41,42^, and eosinophil counts (cells/mL) measured by ADVIA2120 system^®^ (Siemens; Munich, Germany). Results were categorized according to Division of AIDS (DAIDS^43^) age-adjusted reference values for Hb, WBC, lower limit of platelet counts, and ANC; age-adjusted hospital laboratory reference values were used for ALC^44^, and standard hospital laboratory reference values for upper limit platelet counts (500,000 cells/mL for all ages^45^), AMC (normal range: 100–700 cells/mL), and eosinophil counts (normal value: < 500 cells/mL). C-reactive protein (CRP; mg/L) was determined by CRP-Vario^®^ assay read on an ARCHITECT-ci8000^®^ analyzer (Abbott; Wiesbaden, Germany; normal value: < 15 mg/L), and erythrocyte sedimentation rate (ESR, in mm in the 1st hour; normal value: < 15 mm) by photometrical capillary stopped flow kinetic analysis with the Roller 20^®^ analyzer (Alifax SPA; Padova, Italy).
ii. Technical: the time intervals (in days) between blood collection and QFT-GIT analysis (i.e. pre-analytical delay).
iii. Environmental: The season of the year when QFT-GIT assays were performed (winter: December 21–March 20; spring: March 21–June 22; summer: June 23–September 20; and autumn: September 21–December 20); and the local median monthly temperature (in degrees Celsius [°C]; data were obtained from Observatori Fabra, available at: http://www.fabra.cat/meteo/resums/resums.html).

In a subset of patients for whom frozen serum samples taken at the initial assessment were available, serum cotinine concentrations were measured with the Cotinine Direct^®^ ELISA Kit (EIA-5496; DRG International Inc.; Springfield, NJ). The established range of serum cotinine to define secondhand smoke exposure used in most publications is 0.05–10 ng/mL^46^. In the present study TSE was classified according to serum cotinine concentrations as follows: (1) 'no/minimal TSE': < 0.05 ng/mL; (2) 'low TSE': 0.05–2.99 ng/mL; and (3) 'high TSE': ≥ 3 ng/mL^47^.

Statistical analyses were carried out using SPSS version 21.0 (IBM Corp., Armonk, NY, USA). Categorical variables are reported as proportions, and continuous variables as medians with interquartile ranges (IQRs). In bivariate analysis, patients with determinate (i.e. positive or negative) and those with indeterminate QFT-GIT results were compared by means of chi-square tests and Fisher’s exact tests for qualitative variables, and Mann–Whitney *U* and Kruskal–Wallis tests for quantitative variables. Potential correlations between variables were assessed with Spearman's Rho tests. Variables with *p*-values < 0.10 without collinearity were included in subsequent binary logistic regression and multivariate linear regression analysis. Cotinine was excluded from these particular analyses, as results were only available in a subset of the study population. Statistical significance was defined as a *p*-value < 0.05.

## Supplementary Information

Supplementary Information.

## Data Availability

The data underlying this article will be shared on reasonable request to the corresponding author.
