# Supplementary material for: Host, technical, and environmental factors affecting QuantiFERON-TB Gold In-Tube performance in children below 5 years of age
Source: Sci Rep. 2022 Nov 19;12:19908. doi: 10.1038/s41598-022-24433-w (PMC9675832; doi:10.1038/s41598-022-24433-w)
Supplement: Supplementary file 1 — Supplementary Information. [file 41598_2022_24433_MOESM1_ESM.pdf]

Suppl. Table S1. Demographic characteristics, reason for assessment, and QFT-GIT results in the study cohort according to final diagnosis. Data shown as number (%) or median (IQR); the *p*-values refer to statistical comparisons between the 3 diagnostic groups.

	**Uninfected** n=169 (82.8)	**LTBI** n=11 (5.4)	**TB**[†] n=24 (11.8)	***p***	**Entire cohort** n=204
**Demographics**					
Female	83 (49.1)	8 (72.7)	13 (54.2)	0.299	104 (50.9)
Age (months)	24 (10-38)	53 (44-59)	31 (11-37)	**<0.001**	26 (11-40)
<2 years of age	85 (50.3)	0	9 (37.5)	**0.003**	94 (46.1)
Born in Spain	140 (82.8)	7 (63.6)	23 (95.8)	0.055	170 (83.3)
Born to an immigrant family[*]	99 (58.6)	8 (72.7)	15 (62.5)	0.624	122 (59.8)
BCG-vaccinated	13 (7.7)	3 (27.3)	0	0.075	16 (7.8)
**Reason for assessment**				**0.008**	
Suspected TB	32 (18.9)	0	11 (45.8)		43 (21.1)
Contact tracing	114 (67.4)	8 (72.7)	12 (50.0)		134 (65.7)
New entrant screening	23 (13.6)	3 (27.3)	1 (4.2)		27 (13.2)
**QFT-GIT result**				**<0.001**	
Positive	0	7 (63.6)	19 (79.2)		26 (12.7)
Negative	156 (92.3)	4 (36.4)	4 (16.7)		164 (80.4)
Indeterminate	13 (7.7)	0	1 (4.2)		14 (6.8)

Abbreviations: LTBI, latent tuberculosis infection; TB, tuberculosis.
[*]Family origins: Spain, n=82 (40.2%); other European countries, n=4 (1.9%); South America, n=39 (19.2%); Africa, n=47 (23.0%); and Asia, n=32 (15.7%).
[†] Nine out of 24 (37.5%) cases of TB were microbiologically-confirmed.

Suppl. Table S2. Summary of baseline characteristics, tuberculin skin test results, environmental parameters and laboratory results of patients with indeterminate QFT-GIT results.

**Patient**	1	2	3	4	5	6	7	8	9	10	11	12	13	14
**Diagnosis**	TB	U	U	U	U	U	U	U	U	U	U	U	U	U
**Sex**	Male	Male	Male	Male	Male	Female	Female	Female	Female	Female	Female	Male	Male	Male
**Age** (months)	37	1	2	25	43	5	10	7	6	14	21	18	15	20
**Immigrant**	No	No	No	No	No	Yes	Yes	No	No	No	No	No	No	No
**Family origin**	Spain	Bolivia	Spain	Spain	Equador	Ethiopia	Ethiopia	Spain	Spain	Brazil	Spain	Morocco	Spain	Nigeria
**BCG status**	No	No	No	No	No	Unknown	Unknown	No	No	No	No	No	No	No
**Test indication**	CS	CT	CT	CT	CS	NES	NES	CT	CT	CT	CS	CS	CT	CS
**TST** (mm)	5	0	0	0	0	0	0	0	0	0	0	0	0	0
**Season**	winter	autumn	spring	summer	autumn	spring	summer	autumn	spring	summer	winter	winter	spring	autumn
**Median Tª** (ºC)	6.8	17.3	15.3	22.6	16.7	17.9	26.0	17.3	15.3	21.7	9.0	8.2	19.1	12.6
**Hb** (g/dL)	10.1	14.0	11.2	12.3	12.4	12.1	13.5	10.2	-	11.1	-	10.9	12.4	-
**Platelets** (cells/mL)	326.0	532.0	475.0	205.0	322.0	540.0	655.0	370.0	-	335.0	-	386.0	272.0	-
**WBC** (cells/mL)	7.7	11.1	11.2	6.6	19.3	12.5	14.0	14.3	-	10.3	-	11.6	10.0	-
**ALC** (cells/mL)	2.7	3.4	6.3	3.7	1.4	6.9	9.6	5.8	-	4.5	-	7.0	5.4	-
**ANC** (cells/mL)	4.0	5.4	2.7	2.2	17.8	4.1	2.3	6.7	-	5.4	-	3.0	3.4	-
**AMC** (cells/mL)	0.5	1.5	1.4	0.4	0.4	0.7	0.9	1.1	-	1.5	-	1.5	0.5	-
**Eosinophils** (cells/mL)	0.2	0.5	0.3	0.1	0	0.4	0.4	0.3	-	0	-	0.1	0.3	-
**NLR**	1.48	1.59	0.43	0.59	12.7	0.59	0.24	1.16	-	0.98	-	0.43	0.63	-
**MLR**	0.19	0.44	0.22	0.11	0.29	0.10	0.09	0.19	-	0.20	-	0.21	0.09	-
**PLR**	197.0	96.0	75.0	55.0	230.0	78.0	68.0	64.0	-	74.0	-	55.0	50.0	-
**ESR** (mm)/**CRP** (mg/L)	25.0/20.3	2.0/2.7	5.0/1.3	2.0/0.5	18.0/89.8	9.0/-	2.0/-	5.0/3.5	-/-	15.0/14.2	-/51.2	35.0/11.8	8.0/1.3	-/11.3

Abbreviations: ALC, absolute lymphocyte counts; AMC, absolute monocyte counts; ANC, absolute neutrophil counts; CS, clinical suspicion; CT, contact tracing; Hb, hemoglobin levels; MLR, monocyte/lymphocyte ratio; NES, new-entrant screening; NLR, neutrophil/lymphocyte ratio; Tª, median temperature in the month the assay was performed; TB, tuberculosis; TST, tuberculin skin test; U, uninfected; WBC, absolute white blood cell counts.

Suppl. Figure S3. Correlation between average monthly temperatures and background-corrected TB-antigen-induced IFN-γ concentrations in the study participants with positive QFT-GIT results. The values shown are the Spearman correlation coefficient (r) and the corresponding *p* value.

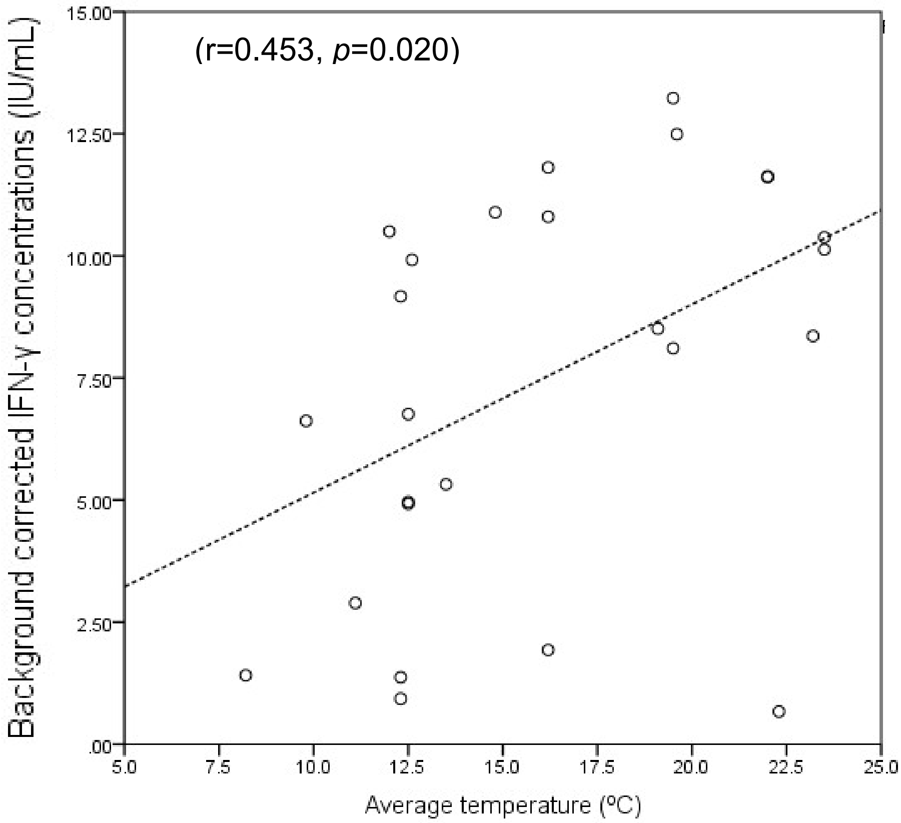

Suppl. Figure S4. Background corrected TB-antigen-induced IFN-γ concentrations in study participants with positive QFT-GIT results according to the season when the test was performed. The values shown are medians and their corresponding interquartile range (IU/mL).

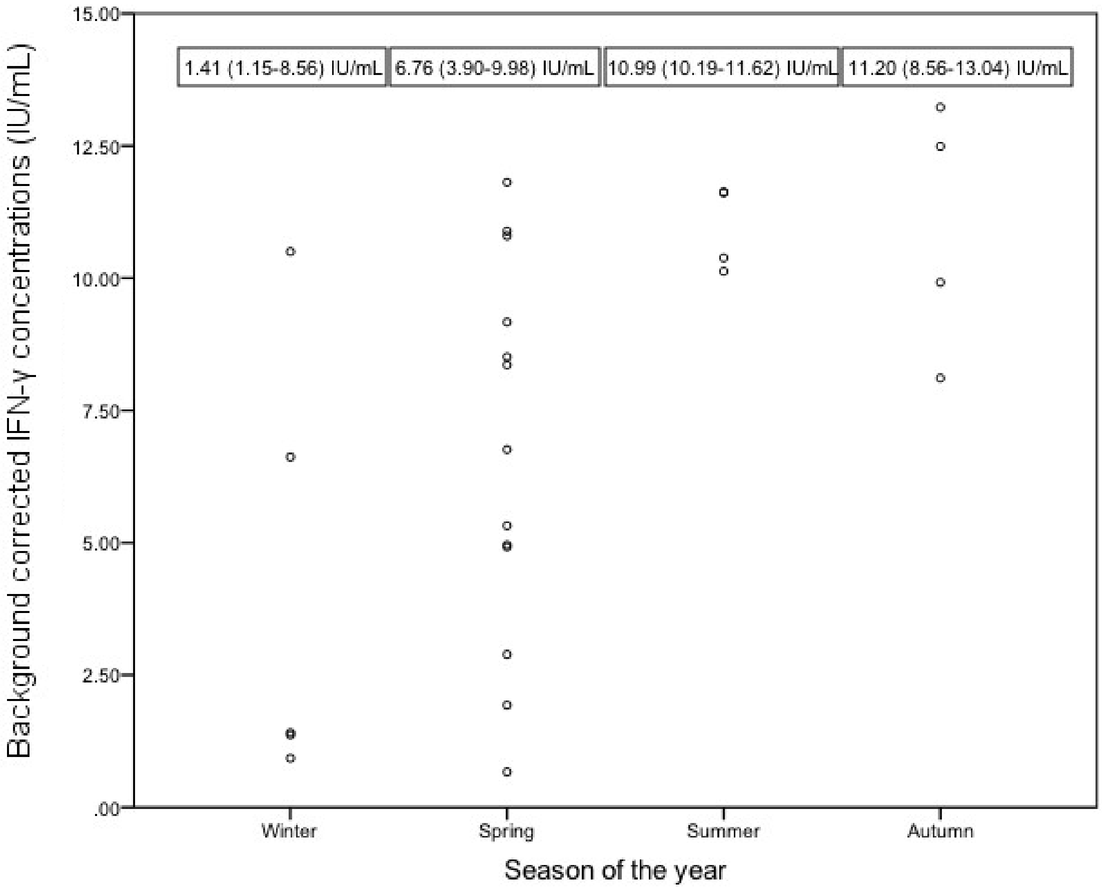

Suppl. Table S5. Serum cotinine concentration and classification of tobacco smoke exposure (TSE) according to final diagnosis. Data shown as number (percentage) or median (interquartile range); the *p*-values refer to comparisons between the 3 diagnostic groups.

	**Uninfected** n=20 (48.8)	**LTBI** n=9 (21.9)	**TB**[†] n=12 (29.3)	***p* value**	**Entire cohort** n=41
Cotinine (ng/mL)	0.83 (0.35-1.95)	0.63 (0.97-1.43)	0.89 (0.31-2.40)	0.576	0.77 (0.26-1.86)
No/minimal TSE	2 (10.0)	2 (22.2)	1 (8.3)	0.663	5 (12.2)
Low TSE	15 (75.0)	7 (77.8)	9 (75)		31 (75.6)
High TSE	3 (15.0)	0	2 (16.7)		5 (12.2)

LTBI: Latent tuberculosis infection; TB: tuberculosis; TSE: tobacco smoke exposure
